# ITGA2, LAMB3, and LAMC2 may be the potential therapeutic targets in pancreatic ductal adenocarcinoma: an integrated bioinformatics analysis

**DOI:** 10.1038/s41598-021-90077-x

**Published:** 2021-05-18

**Authors:** Shajedul Islam, Takao Kitagawa, Byron Baron, Yoshihiro Abiko, Itsuo Chiba, Yasuhiro Kuramitsu

**Affiliations:** 1 Advanced Research Promotion Center, Health Sciences University of Hokkaido, 1757 Kanazawa, Ishikari-Tobetsu, Hokkaido 061-0293 Japan; 2 Centre for Molecular Medicine and Biobanking, University of Malta, Msida, MSD 2080 Malta; 3 Division of Oral Medicine and Pathology, Department of Human Biology and Pathophysiology, School of Dentistry, Health Sciences University of Hokkaido, 1757 Kanazawa, Ishikari-Tobetsu, Hokkaido 061-0293 Japan; 4 Division of Disease Control and Molecular Epidemiology, Department of Oral Growth and Development, School of Dentistry, Health Sciences University of Hokkaido, 1757 Kanazawa, Ishikari-Tobetsu, Hokkaido 061-0293 Japan

**Keywords:** Cancer, Bioinformatics, Gene expression analysis

## Abstract

Pancreatic ductal adenocarcinoma (PDAC) is the most common form of pancreatic cancer with an abysmal prognosis rate over the last few decades. Early diagnosis and prevention could effectively combat this malignancy. Therefore, it is crucial to discover potential biomarkers to identify asymptomatic premalignant or early malignant tumors of PDAC. Gene expression analysis is a powerful technique to identify candidate biomarkers involved in disease progression. In the present study, five independent gene expression datasets, including 321 PDAC tissues and 208 adjacent non-cancerous tissue samples, were subjected to statistical and bioinformatics analysis. A total of 20 differentially expressed genes (DEGs) were identified in PDAC tissues compared to non-cancerous tissue samples. Gene ontology and pathway enrichment analysis showed that DEGs were mainly enriched in extracellular matrix (ECM), cell adhesion, ECM–receptor interaction, and focal adhesion signaling. The protein–protein interaction network was constructed, and the hub genes were evaluated. Collagen type XII alpha 1 chain (COL12A1), fibronectin 1 (FN1), integrin subunit alpha 2 (ITGA2), laminin subunit beta 3 (LAMB3), laminin subunit gamma 2 (LAMC2), thrombospondin 2 (THBS2), and versican (VCAN) were identified as hub genes. The correlation analysis revealed that identified hub genes were significantly interconnected. Wherein COL12A1, FN1, ITGA2, LAMB3, LAMC2, and THBS2 were significantly associated with PDAC pathological stages. The Kaplan–Meier survival plots revealed that ITGA2, LAMB3, and LAMC2 expression were inversely correlated with a prolonged patient survival period. Furthermore, the Human Protein Atlas database was used to validate the expression and cellular origins of hub genes encoded proteins. The protein expression of hub genes was higher in pancreatic cancer tissue than in normal pancreatic tissue samples, wherein ITGA2, LAMB3, and LAMC2 were exclusively expressed in pancreatic cancer cells. Pancreatic cancer cell-specific expression of these three proteins may play pleiotropic roles in cancer progression. Our results collectively suggest that ITGA2, LAMB3, and LAMC2 could provide deep insights into pancreatic carcinogenesis molecular mechanisms and provide attractive therapeutic targets.

## Introduction

Pancreatic ductal adenocarcinoma (PDAC) is the most aggressive and common form of pancreatic cancer, accounting for 95% of all pancreatic malignant neoplasms^1^. The 5-year overall survival rate for patients with PDAC is less than 8% despite advances in medical oncology^2^. The poor prognosis of PDAC may be due to the lack of precise molecular biomarkers for early diagnosis and prognosis^3^. Therefore, there is an urgent need for more effective targeted therapies to improve the survival rate of patients with PDAC^4^.

Gene expression microarrays and gene chips are extensively applied to reveal genetic aspects of diseases. These techniques are routinely used to monitor genome-wide expression levels of genes and are particularly suitable for screening differentially expressed genes (DEGs) between two samples^5^. The identification of DEGs may elucidate cancer pathogenesis, provide early diagnosis, and improve treatment. Hence, gene expression microarray analysis could be a promising approach to identify candidate biomarkers involved in disease progression.

The gene expression profiles from diverse microarray platforms are submitted to several public databases, including Gene Expression Omnibus (GEO: https://www.ncbi.nlm.nih.gov/gds/). Several previous studies used gene expression microarray technology to underpinning the DEGs of PDAC in recent years^6–8^. However, the results were inconsistent, and various aspects remain unclear due to sample heterogeneity. Moreover, those studies have not considered ethnic differences, and many studies have proven that ethnic differences may have relevance for disease gene expression profiles^9,10^. The present study aimed to improve DEGs accuracy and reliability in PDAC compared to adjacent non-cancerous tissue samples using several datasets from different ethnicities.

In the current study, gene expression datasets from PDAC were analyzed to identify DEGs. Gene Ontology (GO) and Kyoto Encyclopedia of Genes and Genomes (KEGG) pathway enrichment were performed using an online toolset. Then, the protein interaction networks were constructed and the hub genes were identified and further verified. The identified hub genes may serve as potential diagnostic and prognostic biomarkers and could be a promising approach for the treatment of PDAC. To the best of our knowledge, this analysis is the first to examine the gene expression microarray database in PDAC tissues and adjacent non-cancerous tissue samples, considering different ethnic groups.

## Materials and methods

### Microarray datasets information

PDAC datasets were obtained from the Gene Expression Omnibus, a public functional genomic database containing high-throughput gene expression data, chips, and microarrays. The GEO database was searched using the following criteria: “human-derived pancreatic ductal adenocarcinoma tissues and adjacent non-cancerous tissue samples” (study keyword), “Homo sapiens” (organism), “expression profiling by array” (study type), “tissue” (attribute name), and “sample count” > 50. After a systematic review, five independent PDAC microarray datasets were selected, including GSE62452^11^, GSE28735^12^, GSE15471^13^, GSE62165^14^, GSE102238^15^, with 321 primary tumor samples and 208 adjacent non-cancerous samples. The dataset GSE62452 was based on the GPL6244 platform (HuGene-1_0-st] Affymetrix Human Gene 1.0 ST Array) and included 69 tumor and 61 adjacent non-cancerous tissue samples. The dataset GSE28735 was based on the GPL6244 platform (HuGene-1_0-st] Affymetrix Human Gene 1.0 ST Array) and had 45 matched tumor and adjacent non-cancerous samples.

The GSE15471 dataset was produced using the GPL570 Platform [(HG‐U133_Plus_2) Affymetrix Human Genome U133 Plus 2.0 Array], including 39 matched tumors and adjacent non-cancerous samples. The GSE62165 dataset was based on the GPL13667 Platform [(HG‐U219) Affymetrix Human Genome U219 Array], which contained 118 tumors and 13 adjacent non-cancerous samples. The GSE102238 dataset was based on the GPL19072 Platform [Agilent-052909 CBC_lncRNAmRNA_V3], which included 50 matched tumor and adjacent non-cancerous samples. These five gene expression profiles were respectively from different regions, including North America, Europe, and Asia, thus averting the differences caused by sample heterogeneity of single profiles and revealing universal DEGs that apply to different ethnic groups, as it has been reported that ethnic difference may affect disease-associated gene expression profiles^9,10^. The clinical datasets included 321 tumors and 208 adjacent non-cancerous tissues diagnosed as PDAC (Table 1). Of note, pancreatic tissue samples in microarray datasets were obtained from the patients who underwent surgical resection for PDAC. Subsequently, tissue samples were stored in liquid nitrogen and/or at − 80 °C until further use. Total RNA was extracted from the snap-frozen tissue samples, and further analysis was carried out. The clinicopathological characteristics of the microarray datasets are briefly shown in Supplementary Table 1.

**Table 1:** Characteristics of datasets used in meta-analysis of PDAC tissues vs. adjacent non-cancerous tissues.

Author, year	GEO accession	Region	Platform	Tissue types and sample numbers		
				PDAC	Adjacent	Count
Yang et al. (2016)^11^	GSE62452	USA	GPL6244	69	61	130
Zhang et al. (2012)^12^	GSE28735	USA	GPL6244	45	45	90
Badea et al. (2008)^13^	GSE15471	Romania	GPL570	39	39	78
Janky et al. (2016)^14^	GSE62165	Belgium	GPL13667	118	13	131
Yang et al. (2020)^8^	GSE102238	China	GPL19072	50	50	100

*PDAC* pancreatic ductal adenocarcinoma.

### Identification of DEGs

DEGs between PDAC and adjacent non-cancerous tissue samples were screened by GEO2R (http://www.ncbi.nlm.nih.gov/geo/geo2r)^16^, an online tool that can be used to compare two or more datasets in a GEO series to identify DEGs according to the experimental conditions. Adjusted *p* values (adj. *p*) and Benjamini and Hochberg false discovery rates were employed as criteria for statistically significant genes and to limit false positives. The data normalization was applied for the five datasets (Supplementary Fig. 1). Probe sets with no corresponding gene symbols were removed, while genes with multiple gene probe sets were averaged. Log2 FC (fold change) ≥ 1.5 or ≥ − 1.5 and adj. *p* < *0.01* was considered statistically significant. An online tool (http://www.interactivenn.net) was applied to draw Venn diagrams of the DEGs^17^. Further, heatmap analysis was visualized with the Heatmapper web application^18^. A total of 20 DEGs were identified, which consisted of 19 upregulated genes and 1 downregulated gene.

### External validation of the identified DEGs mRNA expression level

The external validation was done using the Gene Expression Profiling Interactive Analysis tool^19^ (http://gepia2.cancer-pku.cn/#index; last access: 14th February 2021) by comparing transcriptomic data from The Cancer Genome Atlas (TCGA) (pancreatic adenocarcinoma), the TCGA normal and the Genotype-Tissue Expression (GTEx) database. *p* < *0.05* was considered a statistically significant difference.

### GO and KEGG pathway analysis of DEGs

To uncover the functional roles of DEGs, the GO was used to perform enrichment analysis, which covers the cellular component (CC), biological process (BP), and molecular function (MF) of the selected genes^20^. The KEGG is a database that illustrates the selected gene functions and pathways^21^. The Database for Annotation, Visualization, and Integrated Discovery (DAVID: https://david.ncifcrf.gov; last access: 14th February 2021) is a public online bioinformatics database that contains information on functional biological annotations for genes and proteins^20^. The cut-off criteria were selected based on *p* < *0.01*. Enrichment of the GO terms and KEGG pathways were performed for the candidate DEGs using DAVID.

### Establishment of the PPI network and hub gene identification

To further explore the potential interplay among those DEGs, these were mapped to the STRING (https://string-db.org; version 11.0) database^22^ and only interactions that enjoyed a minimum required combined score > 0.4 were set as significant. Subsequently, the protein–protein interaction (PPI) networks were visualized using Cytoscape 3.8.2 (https://cytoscape.org/), an open-source bioinformatics software platform^23^. A combined score of 0.5 and a tissue-specific (pancreas) filter score of 1 was considered for the construction of the PPI network. Subsequently, the MCODE (Molecular Complex Detection) plugin was used to identify hub genes in the constructed network. The standard for selection was set as follows: MCODE scores ≥ 10, degree cut-off = 2, node score cut-off = 0.2, max depth = 100 and k-score = 2^24^.

### Oncomine analysis of hub genes in pancreatic cancer

An independent database, namely Oncomine (https://www.oncomine.org/resource/login.html; last access: 14th February 2021), was used to validate hub gene expression. In the Oncomine database, the gene name “COL12A1”, “FN1”, “ITGA2”, “LAMB3”, “LAMC2”, “THBS2” or “VCAN” was entered. The differential gene analysis module (cancer vs. normal analysis) was selected to retrieve the results. This analysis presented a series of pancreatic cancer studies and related COL12A1, FN1, ITGA2, LAMB3, LAMC2, THBS2, and VCAN mRNA expression in cancer and normal tissues. The filters were set as follows: (1) Gene: COL12A1 or FN1 or ITGA2 or LAMB3 or LAMC2 or THBS2 or VCAN. (2) Analysis type: cancer vs. normal analysis. (3) Cancer type: pancreatic carcinoma. (4) Sample type: clinical specimen. (5) Data type: mRNA. (6) Threshold settings: *p* < *0.01*; FC > 2; gene rank, top 10%.

### Finding prognostic genes for PDAC

To explore the expression correlation of hub genes in PDAC, the Spearman coefficient correlation was analyzed using the GEPIA2 tool^19^. The interaction efficiency was represented as an R score. An R score of > 0.8 was considered a significant correlation. Next, the expression levels of hub genes and pathological stages in PDAC tissues were assessed using the GEPIA2 platform. The GEPIA2 was also utilized for overall survival and disease-free survival analyses of the hub genes using the TCGA and GTEx databases. The plots were considered significant when showed in both overall and disease-free survival states. Beta-actin was used to normalize the expression of genes, and the median was selected for group cut-off criteria. *p* < *0.05* was considered to indicate a statistically significant difference. Further, the expression of proteins encoded by hub genes in pancreatic cancer was validated using the Human Protein Atlas (HPA: https://www.proteinatlas.org) website based on spatial proteomics data and quantitative transcriptomics data (RNA-Seq) obtained from the immunohistochemical analysis of tissue microarrays^25^.

### Literature review of bioinformatics studies associated with pancreatic cancer

PubMed and Scopus databases were searched to explore existing bioinformatics studies in pancreatic cancer (last access: 15th April 2021). The following criteria were set for PubMed: (pancreatic ductal carcinoma [MeSH Terms]) OR (pancreatic cancer [MeSH Terms]) OR (pancreatic neoplasm [MeSH Terms]) AND (bioinformatics [MeSH Terms]) AND (microarray analysis [MeSH Terms]). For Scopus the following criteria were used: TITLE-ABS-KEY (pancreatic AND ductal AND adenocarcinoma OR pancreatic AND cancer OR pancreatic AND neoplasm AND bioinformatics AND microarray AND analysis). Peer-reviewed studies were considered for the last 10-years, and after a comprehensive analysis, nine studies were selected^6–8,26–31^.

## Results

### Identification of DEGs in PDAC

**Figure 1:**
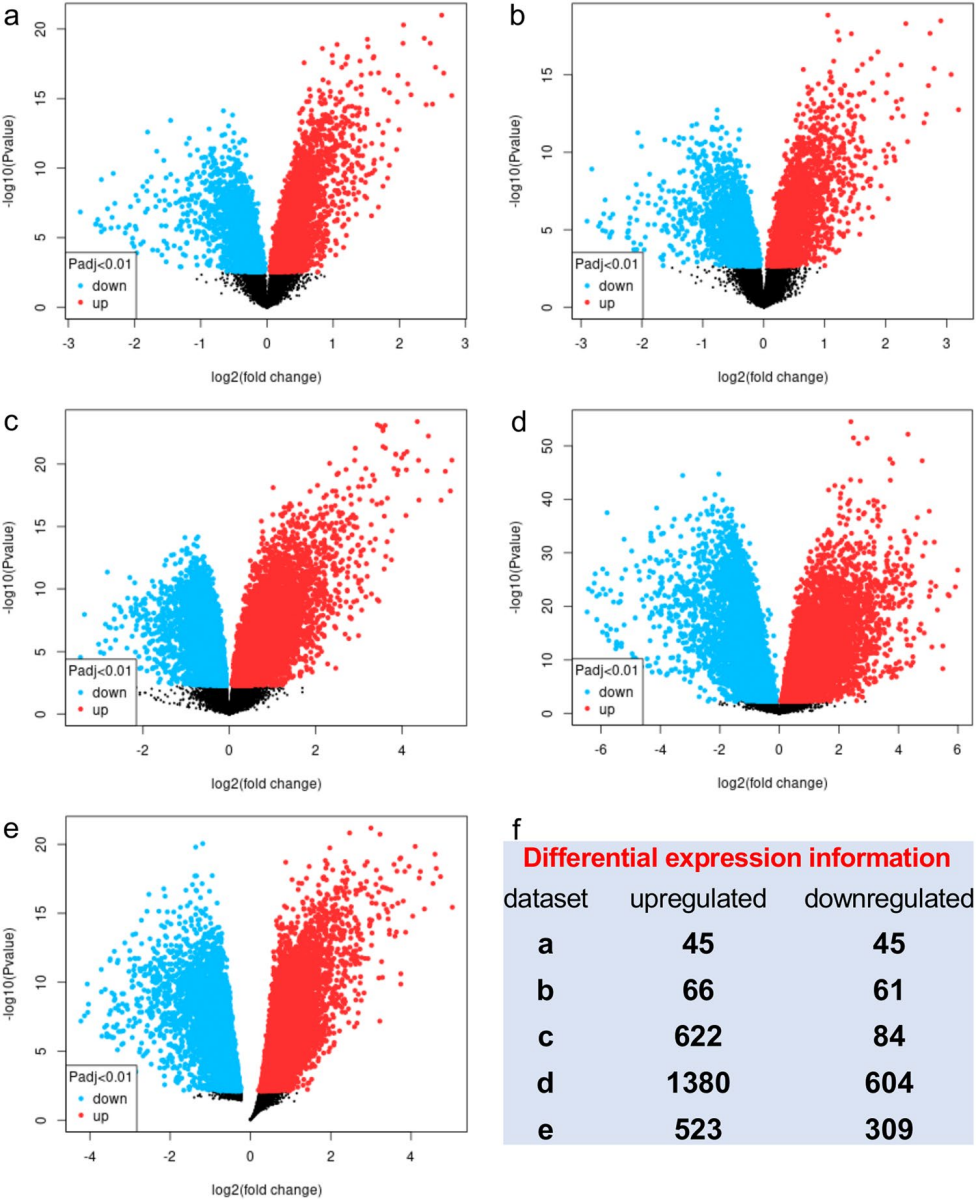
Differential expression of genes between PDAC tissue and adjacent non-cancerous tissue samples in the datasets. (**a**) GSE62452; (**b**) GSE28735; (**c**) GSE15471; (**d**) GSE62165; (**e**) GSE102238. The x-axis indicates the fold-change (log-scaled); the y-axis indicates the *p*-values (log-scaled). The red data-points represent upregulated genes, while blue data-points represent downregulated genes. The black data-points represent genes with no significant difference in expression. (**f**) The differential genes screened based on |Log2FC|≥ 1.5/− 1.5 and a corrected *p* value of < *0.01*. *FC* fold change.

**Figure 2:**
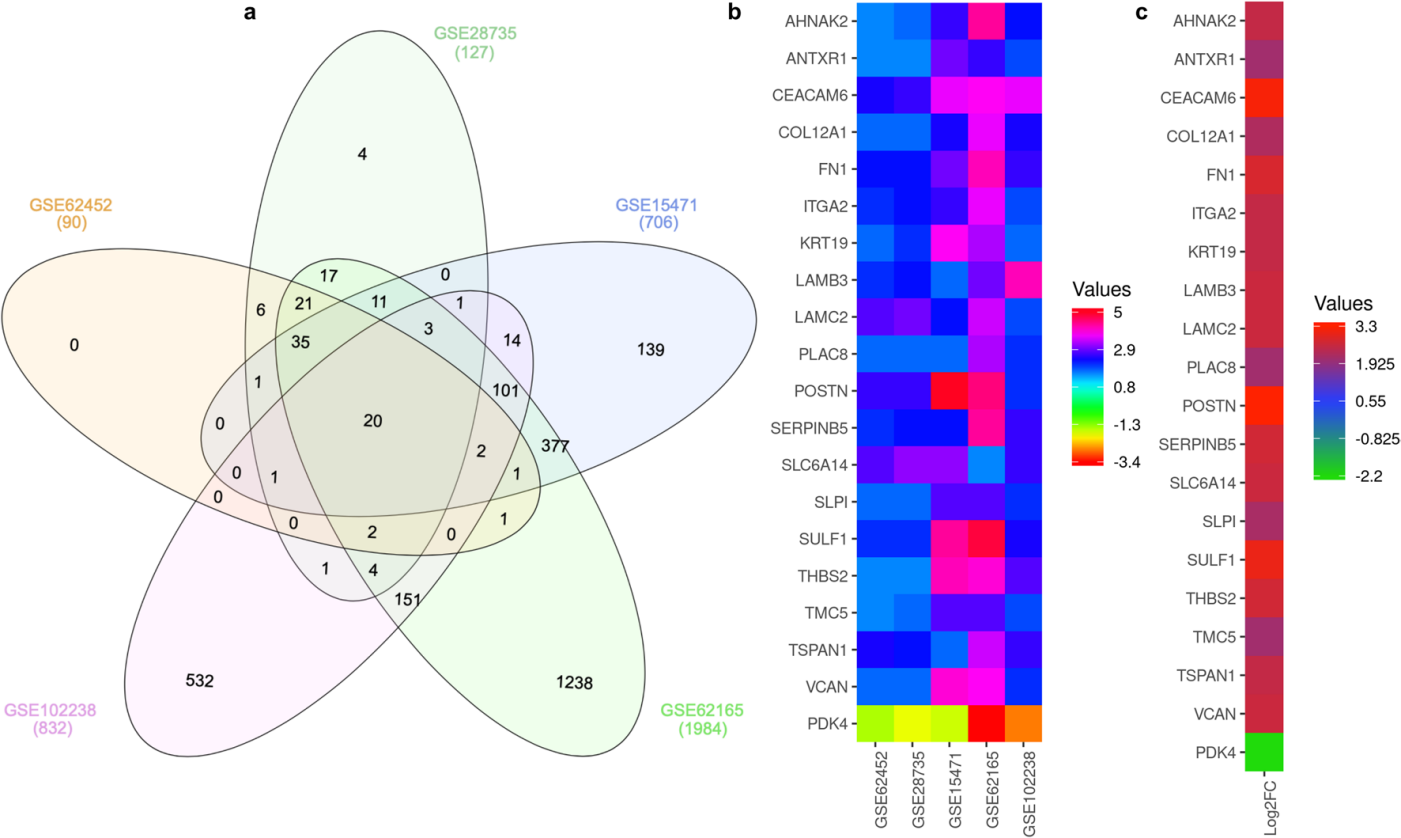
Identification of DEGs. (**a**) Venn diagram of the DEGs in the five datasets. A total of 20 DEGs were included in all five datasets. (**b**) LogFC heatmap image of the 20 commonly changed DEGs of the five datasets. The 5 GEO datasets are denoted on the abscissa, and the gene names are displayed on the ordinate. (**c**) The average LogFC expression values of 20 DEGs. *DEG* differentially expressed gene, *FC* fold change.

The five gene expression microarray datasets for PDAC, GSE62452, GSE28735, GSE15471, GSE62165, and GSE102238, were obtained from GEO. By screening the data with the GEO2R using *p* < *0.01* and log2FC ≥ 1.5 or ≥ − 1.5 as cut-off criteria, 2636 upregulated and 1103 downregulated genes were obtained. In brief, 90 DEGs, including 45 upregulated and 45 downregulated genes, were obtained in the GSE62452 expression profile data (Fig. 1a). GSE28735, 127 DEGs, including 66 upregulated and 61 downregulated genes, were identified (Fig. 1b). In GSE15471, 706 DEGs, including 622 upregulated and 84 downregulated genes, were identified (Fig. 1c). 1984 DEGs, including 1380 upregulated and 604 downregulated genes, were identified from GSE62165 (Fig. 1d). In addition, 832 DEGs, including 523 upregulated and 309 downregulated genes, were identified from GSE102238 (Fig. 1e). The overview of the DEGs results was briefly presented in Fig. 1f. After a comprehensive analysis of the five datasets, 20 DEGs were identified that were differentially expressed in all of them, with 19 genes up-regulated and 1 down-regulated in PDAC tissues compared to adjacent non-cancerous tissues (Fig. 2a). Figure 2b,c provides a heatmap of the 20 DEGs based on Log2FC. The functions and the involvement of identified DEGs on PDAC tissues are shown in Table 2.

**Table 2:** Description of differentially expressed genes and functions in pancreatic cancer.

Gene	Full name	Function
**A. Upregulated differentially expressed genes**		
AHNAK2	AHNAK nucleoprotein 2	Promotes malignant progression by inducing EMT
ANTXR1	Anthrax toxin receptor 1	Enhanced CSC renewal and molecular properties
CEACAM6	Carcinoembryonic antigen-related cell adhesion molecule 6	Promotes malignant progression by inducing EMT; Induction of immune suppression
COL12A1	Collagen type XII alpha 1 chain	Promotes cell migration by remodeling of the ECM
FN1	Fibronectin	Enhanced invasion and metastasis by degrading ECM
ITGA2	Integrin subunit alpha 2	Promotes progression and immune suppression
KRT19	Keratin 19	Promotes malignant progression and associated with poor prognosis
LAMB3	Laminin subunit beta 3	Promotes cell proliferation, invasion, and migration by activating oncogenic pathways
LAMC2	Laminin subunit gamma 2	Degrades ECM and promotes invasion
PLAC8	Placenta associated 8	Promotes cell growth and progression
POSTN	Periostin	Promotes invasion and metastasis
SERPINB5	Serpin family B member 5	Promotes invasion and metastasis
SLC6A14	Solute carrier family 6 member 14	Promotes growth, proliferation, and chemoresistance
SLPI	Secretory leukocyte peptidase inhibitor	Promotes growth, proliferation, and inhibition of apoptosis
SULF1	Sulfatase 1	Promotes invasion and metastasis of tumor
THBS2	Thrombospondin 2	Enhances glycolytic enzymes activity in CSC
TMC5	Transmembrane channel-like 5	Contributes to tumor growth, invasion, and metastasis
TSPAN1	Transmembrane serine protease 4	Promotes invasion and metastasis of tumor
VCAN	Versican	Promotes immune suppression and stromal deposition
**B. Downregulated differentially expressed gene**		
PDK4	Pyruvate Dehydrogenase Kinase 4	Inhibition of EMT

*EMT* epithelial–mesenchymal transition, *CSC* cancer stem-cell, *ECM* extracellular matrix.

### The mRNA expression level of DEGs in PDAC

**Figure 3:**
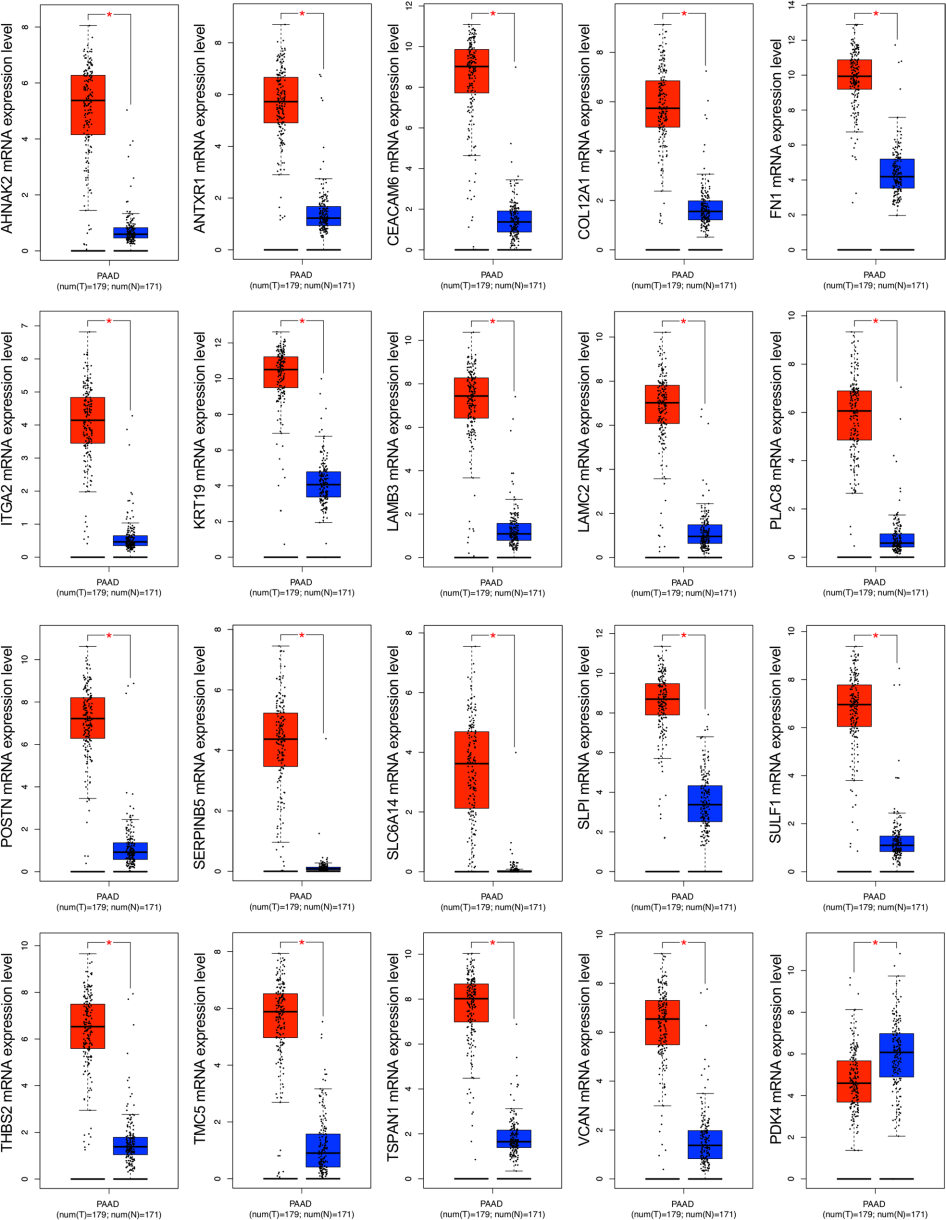
The mRNA expression level analysis of 20 DEGs in PDAC tissues. The boxplots were downloaded from the GEPIA2. The red boxes represent the expression levels in PDAC tissues. In contrast, the blue boxes represent the expression levels in normal tissues. *p* < *0.05* was regarded as statistically significant. *DEGs* differentially expressed genes, *PDAC* pancreatic ductal adenocarcinoma.

To confirm the mRNA expression levels of identified DEGs in PDAC tissues, TCGA datasets were analyzed using the GEPIA2 platform. Boxplots of the DEGs associated with PDAC were downloaded from the GEPIA2. The results demonstrated that upregulated DEGs were significantly overexpressed in PDAC tissues in comparison to normal pancreatic tissues, while the downregulated DEG, PDK4 was significantly reduced in PDAC tissues in comparison to normal pancreatic tissues (*p* < *0.05*) (Fig. 3).

### GO analysis and signaling pathway enrichment of DEGs in PDAC

To elucidate the functions of common DEGs, GO and KEGG pathway enrichment analysis was employed. In the CC category, the upregulated DEGs were mainly enriched in the ECM and extracellular space. In the BP category, the upregulated DEGs were mainly enriched in ECM organization and cell adhesion. While in MF category, upregulated DEGs were enriched with heparin and collagen binding functions. There was no enrichment showed for downregulated DEGs. The ECM–receptor interaction, focal-adhesion, and phosphoinositide-3-kinase-protein kinase B/Akt (PI3K-Akt) signaling were the most enriched pathways for upregulated DEGs. The results of the functional enrichment and KEGG pathway analyses for DEGs are exhibited in Table 3.

**Table 3:** Gene ontology and KEGG pathway analysis of differentially expressed genes.

A. Gene ontology analysis			
Category	GO ID ~ function	Gene count (%)	*p*-value
GOTERM_BP	GO:0030198 ~ extracellular matrix organization	7 (36.8)	3.87E − 08
GOTERM_BP	GO:0007155 ~ cell adhesion	8 (42.1)	2.67E − 07
GOTERM_BP	GO:0035987 ~ endodermal cell differentiation	3 (15.8)	3.75E − 04
GOTERM_BP	GO:0022617 ~ extracellular matrix disassembly	3 (15.8)	0.002951142
GOTERM_BP	GO:0001501 ~ skeletal system development	3 (15.8)	0.009281157
GOTERM_CC	GO:0031012 ~ extracellular matrix	6 (31.6)	1.16E − 05
GOTERM_CC	GO:0005615 ~ extracellular space	9 (47.4)	0.00207988
GOTERM_CC	GO:0070062 ~ extracellular exosome	9 (47.4)	0.00207988
GOTERM_MF	GO:0008201 ~ heparin binding	4 (21.1)	5.15E − 04
GOTERM_MF	GO:0005518 ~ collagen binding	3 (15.8)	0.001632513

B. KEGG pathway analysis			
Pathway ID	Pathway	Gene count (%)	*p*-value
hsa04512	ECM–receptor interaction	5 (26.3)	8.1175E − 07
hsa04510	Focal adhesion	5 (26.3)	2.5476E − 05
hsa05222	Small cell lung cancer	4 (21.1)	6.1498E − 5
hsa04151	PI3K-Akt signaling pathway	4 (21.1)	0.00019292
hsa05146	Amoebiasis	3 (15.8)	0.00469651
hsa05200	Pathways in cancer	4 (21.1)	0.00544945

*ECM* extracellular matrix, *PI3K-Akt* phosphatidylinositol 3-kinase/protein kinase B.

### PPI network construction and identification of hub nodes

**Figure 4:**
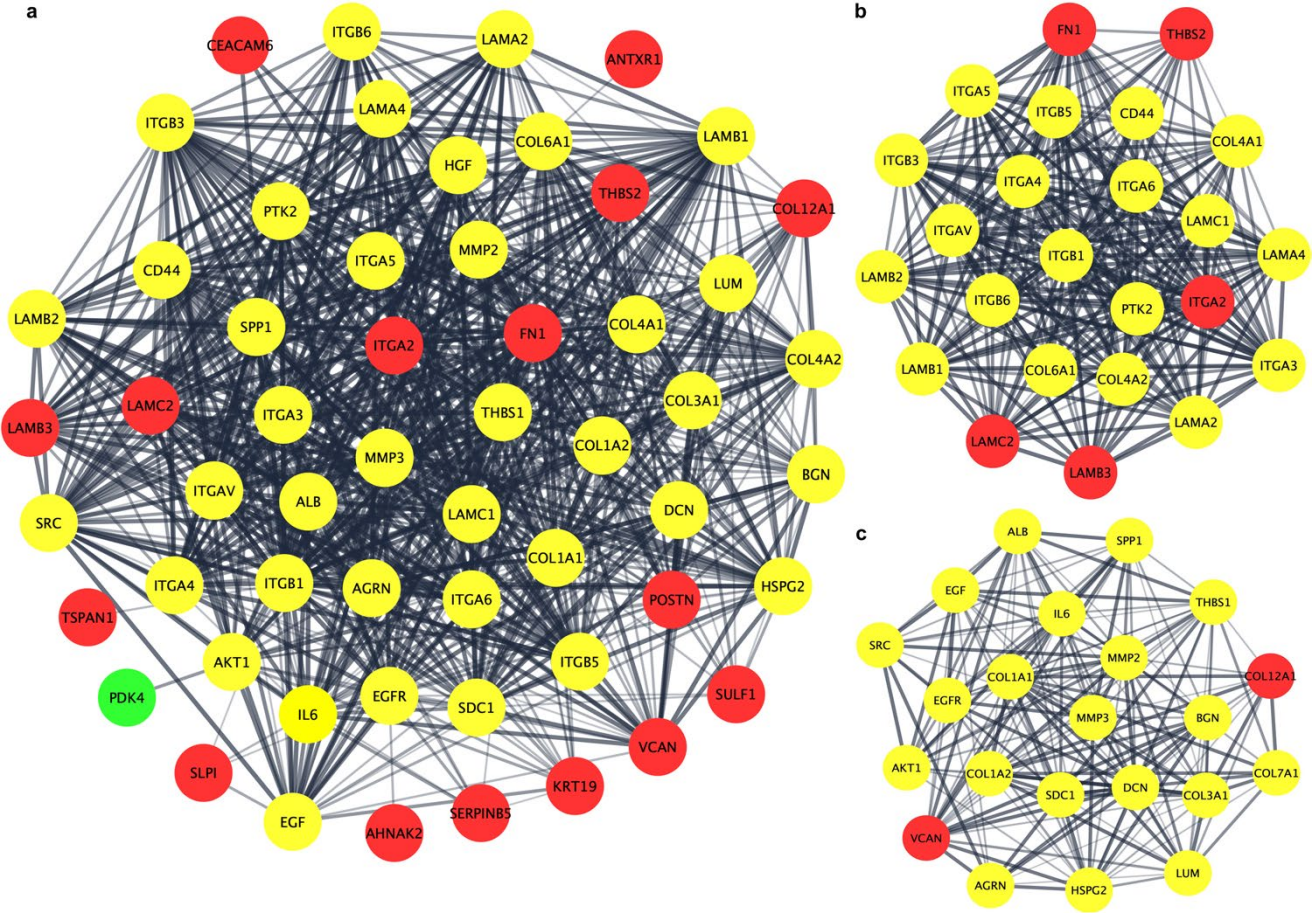
PPI network construction of DEGs and identification of hub genes. (**a**) PPI network was constructed using Cytoscape. Red nodes represent upregulated genes, whereas green nodes represent downregulated genes. The line represents the interaction relationship between nodes. (**b**) Significant modules of cluster-1 were identified from the PPI network via the MCODE plug-in. This module consisted of 5 upregulated genes, which are represented by red color. (**c**) Significant modules of cluster-2 were identified from the PPI network via the MCODE plug-in. This module consisted of 2 upregulated genes, and red nodes represent key genes. *PPI* protein–protein interaction, *DEGs* differentially expressed genes.

The PPI network of the DEGs was constructed using Cytoscape software and the STRING database. The PPI network of DEGs consisted of 58 nodes and 811 edges (Fig. 4a). The Cytoscape tool MCODE was used to screen hub genes in the network, with a cluster score of ≥ 10 as the inclusion criterion. The MCODE modules included 46 nodes and 432 edges with two clusters. Cluster-1 included 24 nodes and 260 edges with a combined score of 22.6. Wherein cluster-2 included 22 nodes and 172 edges with a cluster score of 16.4. After a comprehensive analysis, hub genes were identified from two clusters highlighted in red color (Fig. 4b,c). COL12A1, FN1, ITGA2, LAMB3, LAMC2, THBS2, and VCAN were finally selected as hub genes. The MCODE plugin scores are briefly shown in Table 4.

**Table 4:** MCODE cluster scores on PPI network of differentially expressed genes.

Query term	MCODE score
**A. Cluster-1, combined score: 22.6**	
LAMC2	18.3
LAMB3	18.0
ITGA2	17.4
FN1	17.1
THBS2	16.9
**B. Cluster-2, combined score: 16.4**	
VCAN	14.1
COL12A1	13.8

*MCODE* molecular complex detection, *PPI* protein–protein interaction.

### Oncomine analysis of hub genes in pancreatic cancer databases

**Figure 5:**
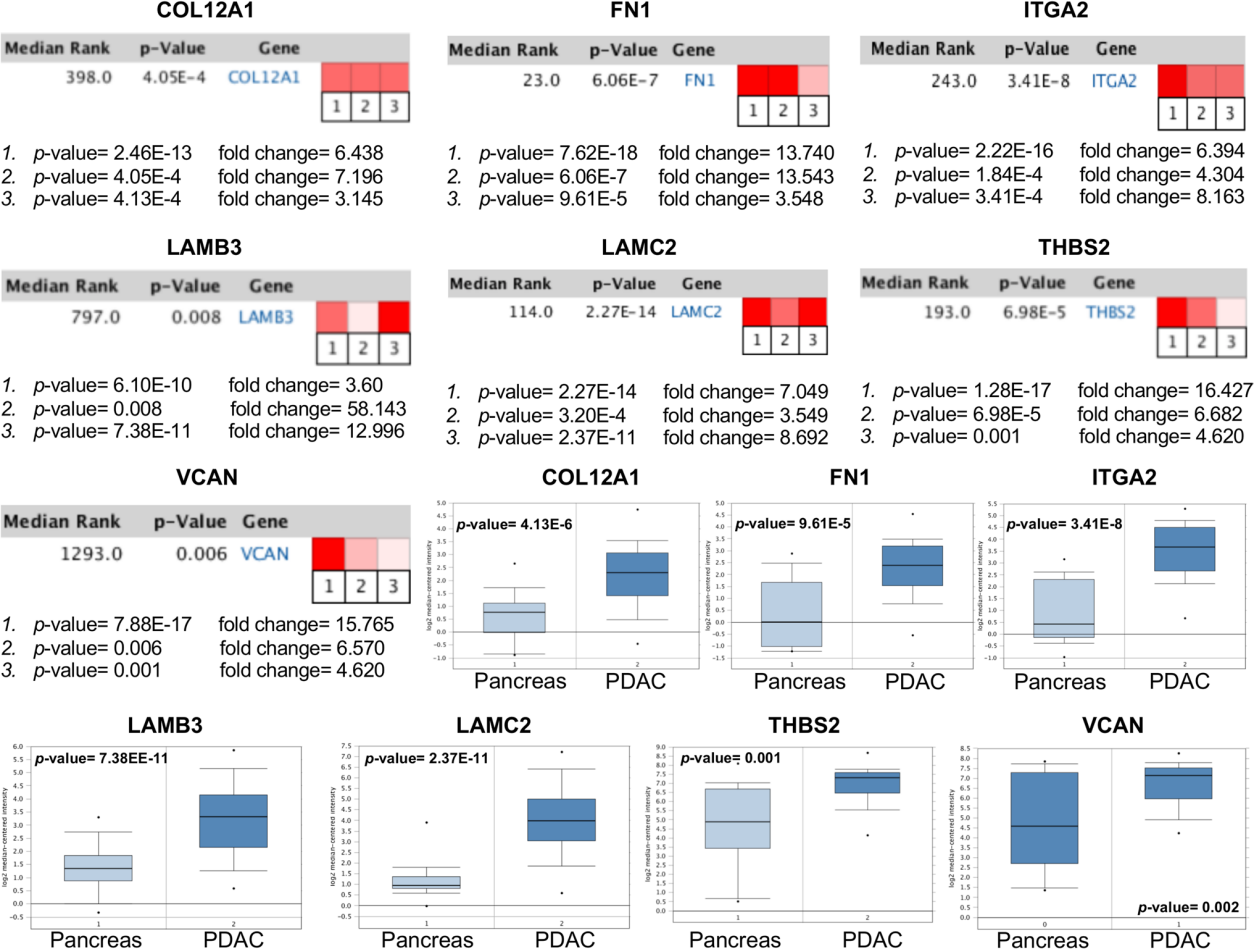
Oncomine analysis of key candidate genes in pancreatic cancer vs. normal tissue. Heat maps of key candidate gene expression in clinical pancreatic cancer samples vs. normal pancreatic tissue samples. *[*1. Pancreatic ductal adenocarcinoma epithelia vs. normal Badea pancreas; 2. Pancreatic ductal adenocarcinoma epithelia vs. normal Lacobuzio-Donahue pancreas; 3. Pancreatic carcinoma vs. normal Pei pancreas). The color depth represents the intensity of fold changes. Box plots represent the mRNA expression level in pancreatic cancer and normal pancreatic tissues in the Pei pancreas dataset. *p* < *0.01* was considered statistically significant.

As COL12A1, FN1, ITGA2, LAMB3, LAMC2, THBS2, and VCAN were selected from the other DEGs, further confirmation of the altered expressions was necessary. Oncomine analysis of cancer vs. normal tissue confirmed that COL12A1, FN1, ITGA2, LAMB3, LAMC2, THBS2, and VCAN were significantly overexpressed in pancreatic cancer from different datasets. A brief overview of those key genes expression in pancreatic cancer was shown by using a heatmap. The color intensity reflects the fold changes between different datasets. Moreover, in the Pei pancreas dataset, COL12A1, FN1, ITGA2, LAMB3, LAMC2, THBS2, and VCAN mRNA expression levels were higher in pancreatic cancer tissue than in normal pancreatic tissue samples (Fig. 5).

### Expression correlation of hub genes in PDAC

**Figure 6:**
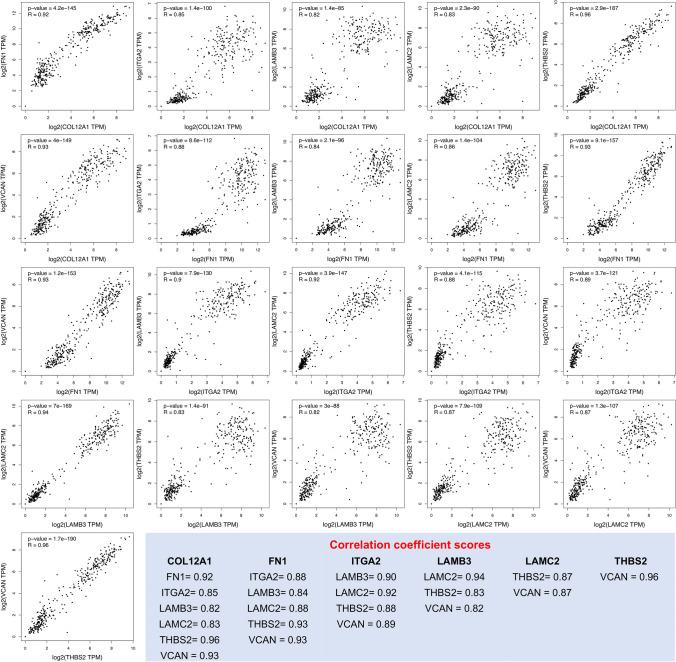
Expression correlation analysis of hub genes in PDAC tissues. The GEPIA2 platform analyzed the expression correlation levels. The Spearman correlation coefficient was used, and an R score of > 0.8 was considered statistically significant. The light blue box represents the correlation coefficient based on R scores.

To explore the correlation among the hub genes in PDAC, TCGA datasets were analyzed using the GEPIA2 platform. COL12A1, FN1, ITGA2, LAMB3, LAMC2, THBS2, and VCAN were observed to be significantly correlated (Fig. 6).

### Association of hub genes in PDAC pathological stages

**Figure 7:**
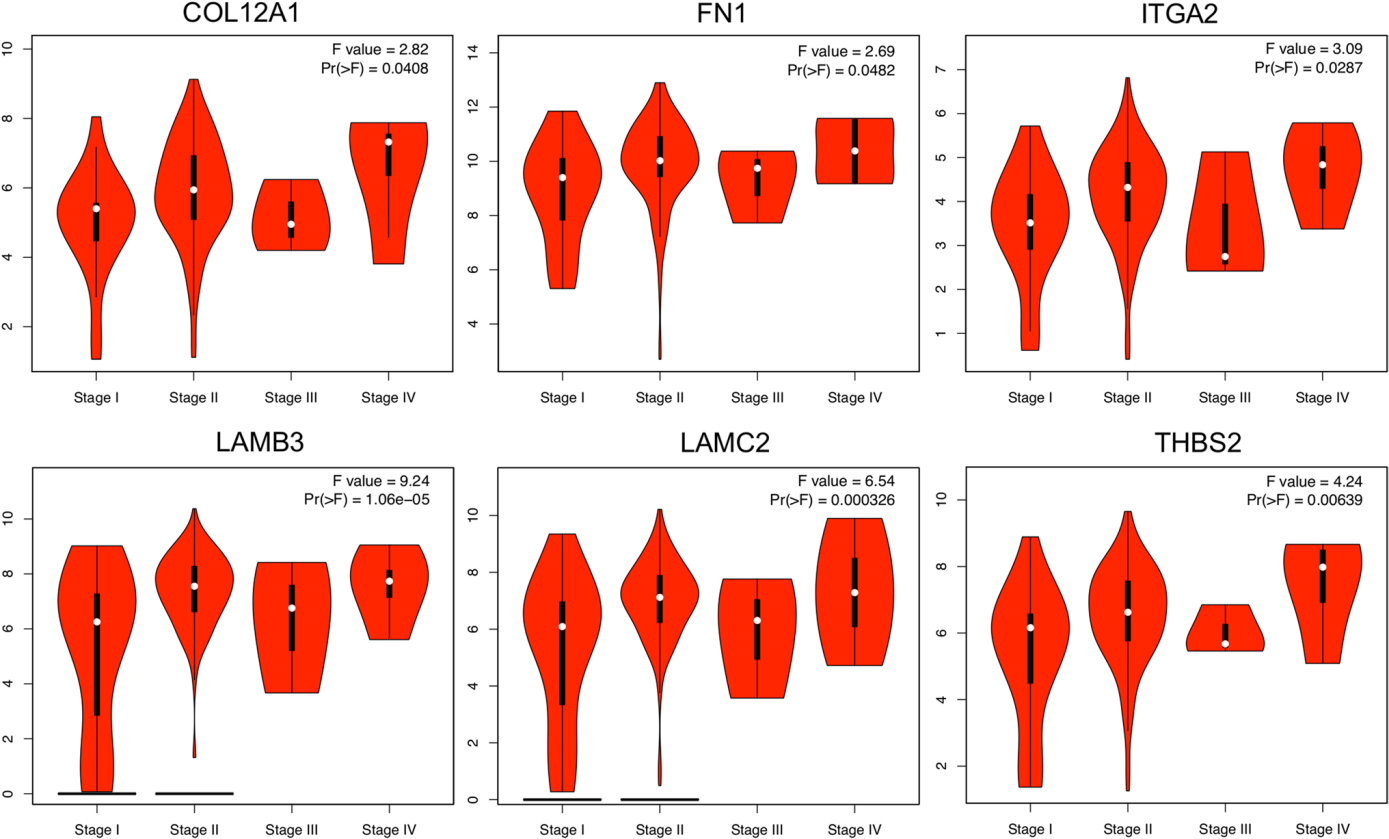
Pathological stages of hub genes in PDAC tissues. Association of mRNA expression and pathological tumor stages in patients with PDAC. Violin plots were created using the GEPIA2 platform based on the TCGA PDAC dataset. F-value indicates the statistical value of the F test; Pr (> F) indicates *p* value. A *p* value of < *0.05* was considered statistically significant.

Further analysis of the TCGA PDAC data in GEPIA2 showed that the hub genes were significantly correlated with the pathological disease stages, underlying their prognostic value for PDAC. COL12A1, FN1, ITGA2, LAMB3, LAMC2, and THBS2 were observed to be significantly associated with PDAC stages (Fig. 7), wherein no significant association on PDAC tumor stages and VCAN was observed (data not shown).

### Survival analysis of hub genes in PDAC

**Figure 8:**
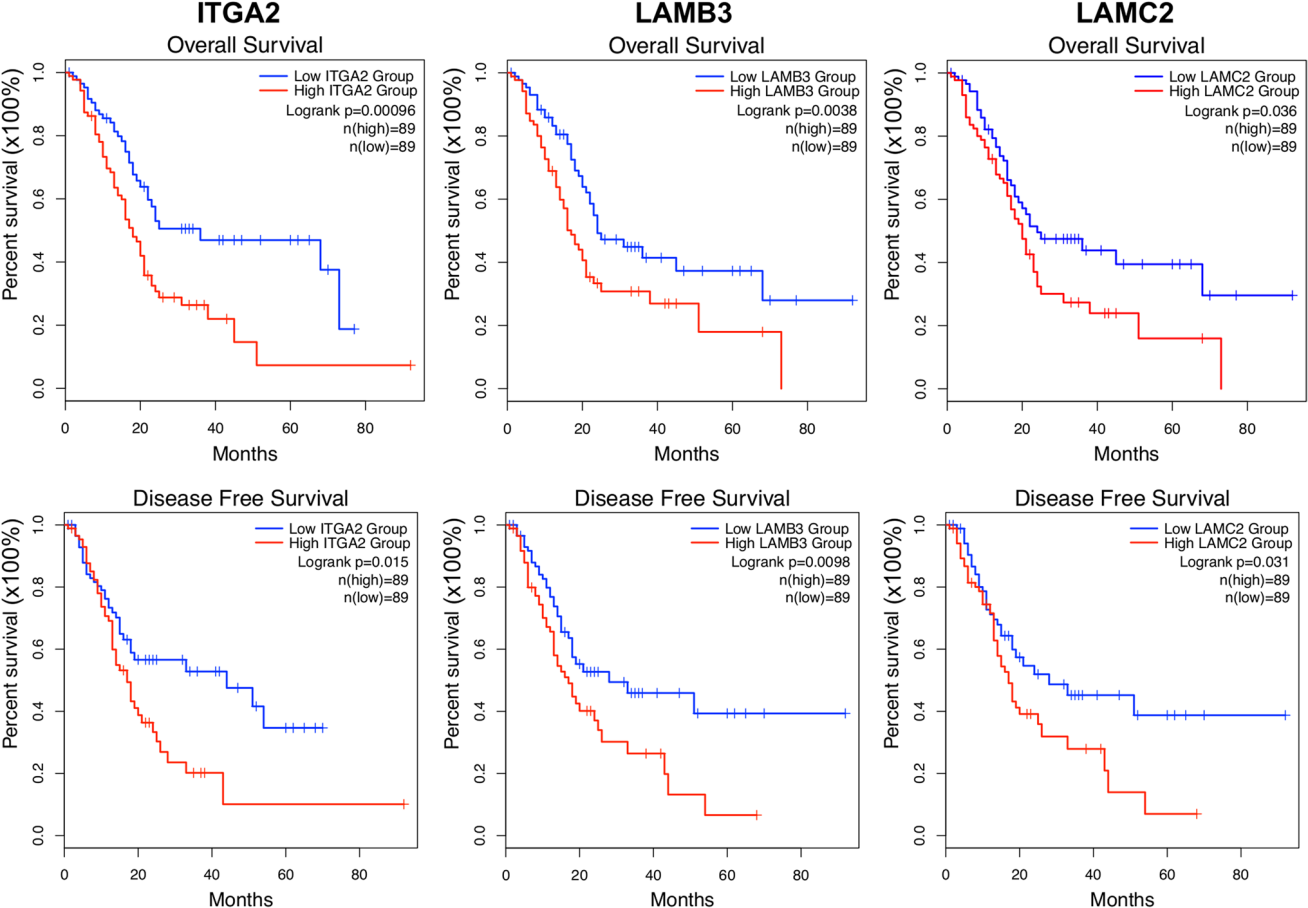
Kaplan–Meier survival plots of hub genes in PDAC tissues. The Kaplan–Meier plots were generated by using the GEPIA2 platform. The overall survival and disease-free survival plots compared a high-risk group (in red) and a low-risk group (in blue) in PDAC tissues. *p* < *0.05* were regarded as statistically significant.

The Kaplan–Meier survival plots were used to observe the overall survival and disease free-survival status of the hub genes in PDAC. Elevated expression levels of ITGA2, LAMB3, and LAMC2 were found to be inversely correlated with prolonged patient survival (Fig. 8), whereas no significant relationship was observed for other genes (data not shown).

### Validation of expression of hub genes-encoded proteins

**Figure 9:**
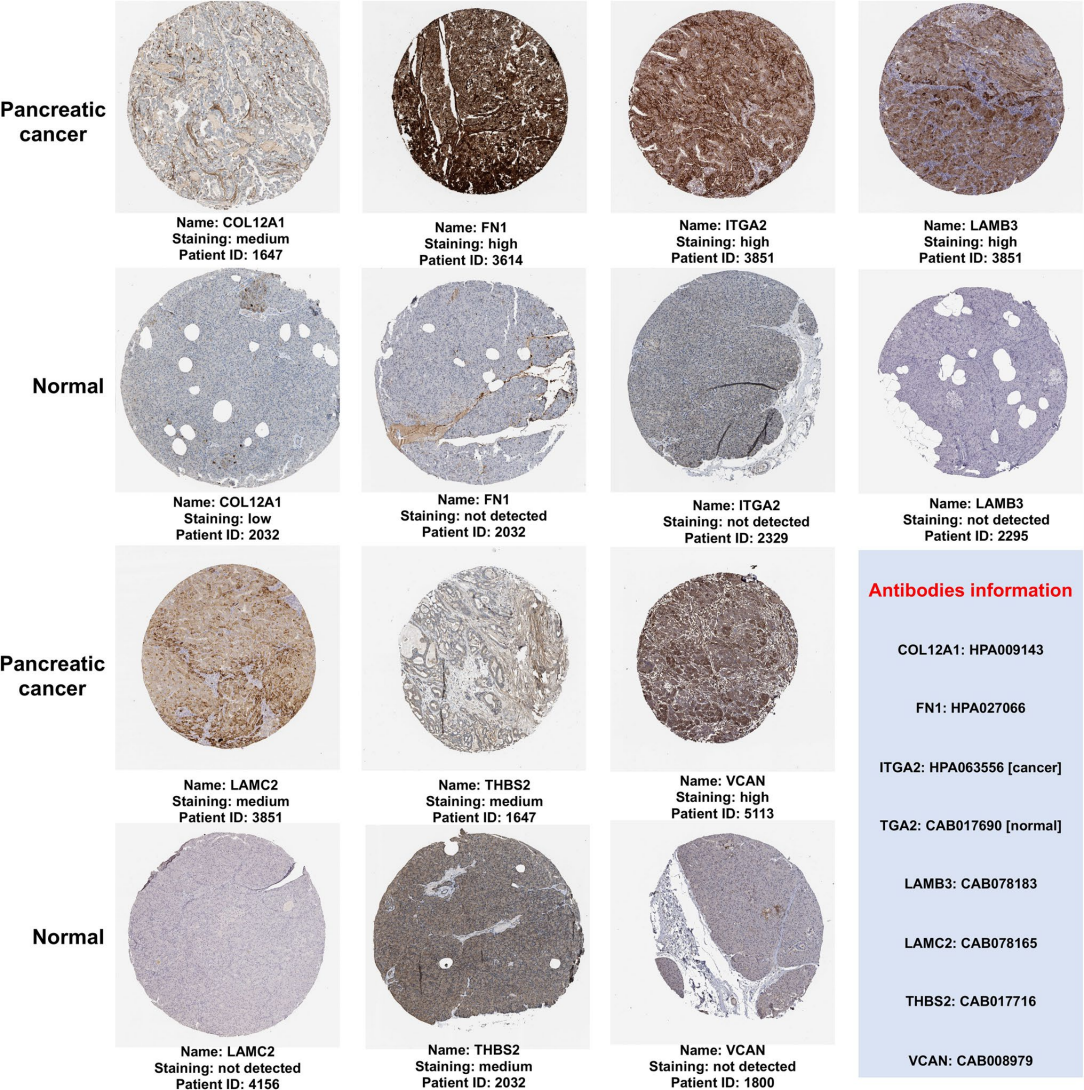
Immunohistochemical expression of hub genes in human pancreatic cancer specimens. The immunohistochemical data were obtained from the Human Protein Atlas. Staining demonstrated that the protein expression of hub genes was higher in pancreatic cancer tissue than in normal pancreatic tissue samples. The light blue box represents antibodies information. Image courtesy: Human Protein Atlas (http://www.proteinatlas.org).

The expression levels of proteins encoded by the COL12A1, FN1, ITGA2, LAMB3, LAMC2, THBS2, and VCAN were obtained. The protein expression profiles in pancreatic cancer clinical specimens are shown in Fig. 9. The antibody intensity for FN1, ITGA2, LAMB3, LAMC2, and VCAN was higher in PDAC tissues, while no staining was observed in corresponding normal tissues. COL12A1 had medium staining intensity with low intensity observed in normal pancreatic tissues. THBS2 had medium staining intensity in both pancreatic cancer and normal pancreatic tissues. Further observations revealed that COL12A1 and FN1 were predominantly expressed by stromal cells. THBS2 and VCAN were expressed in both stromal and pancreatic cancer cells, whereas ITGA2, LAMB3, and LAMC2 were solely expressed by pancreatic cancer cells.

### Identification of hub genes in previous bioinformatics studies associated with pancreatic cancer

The literature review was done to investigate hub genes from previous bioinformatics studies in pancreatic cancer. Nine bioinformatics studies were chosen after a comprehensive analysis based on the criteria which we set. The hub genes, their associated pathways, and potential clinical relevance were explored, which is shown in Table 5. In brief, collagens (COL1A1, COL1A2, COL3A1, COL3A2, and COL5A2), integrins (ITGA2 and ITGB2), laminins (LAMA3, LAMB3, and LAMC2), and fibronectin were the most common hub genes found in those studies. Further, the cell cycle regulation, tissue remodeling, ribosomal protein, and nuclear pore complex-related genes were found to be altered in those studies. The pathways analysis has shown that ECM–receptor interaction, focal adhesion, pathways in cancer, and altered metabolic pathways have been the most commonly involved with those hub genes.

**Table 5:** Literature review of the existing bioinformatics studies associated with pancreatic cancer.

Author, year	Hub genes	Pathways	Observations
Yang et al. (2020)^8^	AHNAK2, CDH3, IFI27, ITGA2, LAMB3, SFN, SLC6A14, and TMPRSS4	Pancreatic secretion, pathways in cancer, p53 signaling pathway, MAPK signaling pathway, Insulin signaling pathway, and pancreatic cancer	AHNAK2, CDH3, IFI27, ITGA2, LAMB3, SLC6A14, and TMPRSS4 may have great diagnostic and prognostic value for pancreatic cancer
Jin et al. (2020)^7^	*TOP2A, CDK1, PRM2, PRC1, NEK2, ZWINNT1, DTL, MELK, CENPF, CEP55, ANLN, ASPM, and ECT2*	ECM–receptor interaction, focal adhesion, pathways in cancer, proteoglycans in cancer, p53 signaling pathway, and PI3-Akt signaling pathway	CDK1, TOP2A, and CEP55 may play pleiotropic roles in the progression of pancreatic cancer
Li et al. (2019)^26^	ALB, COL1A2, EGF, COL3A1, FN1, CEL, ITGA2, COL5A2, MMP1, and CELA3B	Protein digestion and absorption, ECM–receptor interaction, pancreatic secretion, and fat digestion and absorption	COL1A2, COL3A1, and COL5A2 may promote pancreatic fibrosis and EMT via the ECM–receptor interaction pathway in the early stages of pancreatic cancer
Liu et al. (2019)^27^	HN1, ITGA2, S100A6, KIF1A, DYM, and BACE1	Ubiquitin-mediated proteolysis and pathways in cancer	HN1, ITGA2, and S100A6 may be promising potential targets for diagnosing and treating pancreatic cancer
Shen et al. (2018)^31^	*RPL13, RPL17, RPL21, RPL22, RPL23, RPL26, RPL31, RPL35A, RPL36A*,*RPL37, RPL39, RPL7, RPS17,* *RPS23, RPS3A,RPS6,* *RPS7,* *NUP107,* *NUP160, and HNRNPU*	Ribosome pathway and the spliceosome pathway	*Nup170*, *Nup160*, and *HNRNPU* may be used as possible molecular markers for early diagnosis of pancreatic cancer
Pan et al. (2018)^28^	CXCL8, ADCY7, ITGAM, ITGB2, ITGB1, IL1A, ICAM1, ITGA2, THBS2, SDC1, COL3A1, COL1A2, COL1A1, MYL9, LAMA3, LAMB3, LAMC2, COL4A1 and FN1	ECM–receptor interaction, focal adhesion, pathways in cancer, and small cell lung cancer	LAMA3, LAMB3, LAMC2, COL4A1, and FN1 may involve the malignant progression of pancreatic cancer
Tang et al. (2018)^6^	DKK1 and HMGA2	Glycine, serine, and threonine metabolism	DKK1 and HMGA2 may be important in the progression of pancreatic cancer
Li et al. (2018)^29^	ALB, EGF, FN1, ITGA2, COL1A2, SPARC, COL3A1, TIMP1, COL5A1, COL11A1, and MMP7	ECM–receptor interaction, cell adhesion, and transforming growth factor-beta receptor signaling pathway	ITGA2 and MMP7 may act as potential diagnostic and therapeutic biomarkers for pancreatic cancer
Wang et al. (2015)^30^	VCAN, SULF1, COL8A1, FAP, COL1A1, THBS2, CTHRC1, COL1A2, COL6A3, FN1, COL10A1, COL3A1, TIMP, AEBP1, and COL5A1	ECM–receptor interaction, focal adhesion, and complement and coagulation cascades	The collagen family genes and FN1 may play an essential role in the progression of pancreatic cancer

## Discussion

In the present study, 20 DEGs were identified (19 upregulated and 1 downregulated), which were differentially expressed in PDAC tissue compared to the adjacent non-cancerous pancreatic tissue samples. By using an online tool, the mRNA expression levels of DEGs in PDAC tissue samples were validated. The GO and KEGG pathway analysis revealed that DEGs were primarily enriched with ECM-organization, cell adhesion, ECM–receptor interaction, and focal adhesion, especially for the upregulated genes. The PPI network was constructed, and hub genes were selected. COL12A1, FN1, ITGA2, LAMB3, LAMC2, THBS2, and VCAN were identified as hub genes. To verify the expression level of hub genes, an independent database was then used. This confirmed that, compared to normal pancreatic tissues, identified hub genes were highly expressed in pancreatic cancer samples. The correlation analysis revealed that the hub genes in PDAC tissue samples are significantly interconnected. The interaction of hub genes with pathological stages in patients with PDAC showed that the expression of COL12A1, FN1, ITGA2, LAMB3, LAMC2, and THBS2 is negatively associated with disease progression. The survival plots of Kaplan–Meier showed that ITGA2, LAMB3, and LAMC2 expression are inversely correlated with prolonged patient survival. Using histopathological images from the Human Protein Atlas platform, the protein expression profiles of hub genes were validated. It was found that proteins encoded by hub genes are highly expressed in pancreatic cancer tissue compared to normal pancreatic tissue samples. It was also observed that ITGA2, LAMB3, and LAMC2 were the only proteins expressed in pancreatic cancer cells but not in stromal cells. The cancer cells specific expression of these three proteins might be crucial for PDAC pathogenesis and progression. Together, this data suggested that ITGA2, LAMB3, and LAMC2 individually might have high prognostic and diagnostic values, as well as the potential to be therapeutic targets for PDAC.

ITGA2 is a collagen receptor expressed on cell membranes and forms a heterodimer α2β1 with a β subunit, which mediates cell-to-ECM attachment^32^. The increased ITGA2 level was reported in pancreatic cancer and others, including gastric, liver, prostate, and breast cancer^33^. The increased ITGA2 expression promotes pancreatic cancer cell migration, invasion, metastasis, and chemoresistance^34,35^. In contrast, inhibition of ITGA2 abrogated these functions^33^. Although the exact mechanism by which ITGA2 is involved in pancreatic carcinogenesis remains unclear, it has been suggested that ITGA2 promotes pancreatic cancer progression through ECM remodeling^36,37^. The reconstituted ECM triggers pancreatic cancer progression by directly promoting cellular transformation and enhancing tumorigenic microenvironment formation by affecting stromal-cell behavior^38^. In this process, ITGA2 activates fibroblasts to cancer-associated fibroblasts (CAFs), resulting in extensive desmoplasia with ECM deposition^39^, wherein desmoplasia is a characteristic feature of PDAC and constitutes up to 90% of the tumor volume. Mainly ECM and CAF, immune cells, and vascular components form the desmoplastic microenvironment^40,41^. ECM is a three-dimensional structural complex consisting of structural and non-structural proteins^42,43^. ECM-proteins can affect PDAC progression and patient survival by promoting cancer cell proliferation and metastatic spread^44^. Even though stromal cells produce over 90% of the ECM mass in PDAC, cancer cells produce elevated ECM-proteins, and cancer cell-derived ECM-proteins play important roles in PDAC carcinogenesis^45,46^. A previous report suggested that ECM proteins originating from cancer cells were the most strongly connected to poor patient survival. In contrast, ECM-proteins derived from stromal cells, include both proteins linked to good and poor patient outcomes^47^. Hence, using the Human Protein Atlas database, the protein expression profiles and cellular origins of hub genes encoded proteins in pancreatic cancer tissues were observed. ITGA2 is the transmembrane receptor for collagens and related proteins, as mentioned above^32^, while COL12A1, FN1, LAMB3, LAMC2, THBS2, and VCAN are ECM-related proteins^47^.

Our histopathological evidence has shown that COL12A1 and FN1 are expressed from stromal cells, THBS2, and VCAN from stromal and cancer cells, while ITGA2, LAMB3, and LAMC2 are expressed solely from the cancer cells. The Kaplan–Meier survival plots showed that ITGA2, among the ECM-proteins LAMB3 and LAMC2 expression, is inversely correlated with the overall and disease-free survival status in PDAC. Interestingly, a previous report confirmed that LAMB3 and LAMC2 were exclusively derived from pancreatic cancer cells^47^. This study reached a similar conclusion that increased levels of ECM-proteins originated from cancer cells, rather than being solely produced by stromal cells, correlate with poor patient outcomes. However, further studies are needed to clarify this phenomenon. Meanwhile, these results may explain why previous non-selective ECM depletion strategies led to poor patient outcomes and suggest more accurate ECM manipulations as PDAC treatments^48^. Together, the present data and the previous report suggested that cancer-cell-derived ECM-proteins may be potential therapeutic targets^47^. Therefore, sorting out the composition and changes of the ECM during PDAC progression would guide the development and application of more effective PDAC therapies.

It is worth noting that DEGs in PDAC have already been demonstrated in several studies^6–8,26–31^. However, the results were not consistent, which could be due to the differences in the selection of datasets and statistical procedures. Then, using effective search engines, we performed a literature review of existing pancreatic cancer bioinformatics studies and explored hub genes. In brief, the hub genes were mainly involved with ECM remodeling and organization. The predominant expression of collagen, integrin, and laminin family genes was observed in those studies, clarifying their role in ECM remodeling. The reconstituted ECM was reported to promote pancreatic fibrosis and epithelial-mesenchymal transition (EMT) in early stages of PDAC pathogenesis^38^. Thus, ECM manipulation is an appealing therapeutic strategy for PDAC patients.

While the occurrence of PDAC has been observed to differ between racial/ethnic subpopulations, this disparity may be partially explained by the prevalence of risk factors (smoking and drinking alcohol, obesity, diabetes, and family history) among ethnic groups^49,50^. These racial/ethnic variations might result in tumor biology differences in PDAC^50^. Biomarkers that could be useful regardless of racial differences are thus urgently needed. In this study, we selected the datasets from different regions, thus averting the differences caused by the samples heterogeneity and revealing universal DEGs that apply to different ethnic groups. The identified DEGs in this analysis might be applicable irrespective of the ethnicities and may allow the development of more targeted prevention strategies. However, a lack of adequate validation in vitro or in vivo is a limitation of this study. Moreover, due to GEO limitations, the clinicopathological data and demographic variables within this study datasets were not detailed enough. Thus, we failed to consider factors such as the presence of different ethnicities within datasets. Our future research will include experimental verification of this meta-analysis results using different laboratory approaches.

In conclusion, the present meta-analysis identified 20 DEGs. The hub genes are COL12A1, FN1, ITGA2, LAMB3, LAMC2, THBS2, and VCAN. The Kaplan–Meier survival plots indicate that ITGA2, LAMB3, and LAMC2 are inversely correlated with prolonged patient survival. Histopathological evidence shows that ITGA2, LAMB3, and LAMC2 are expressed exclusively from pancreatic cancer cells. The specific expression of these three proteins by cancer cells could make them promising potential targets for diagnosing and treating pancreatic cancer.

## Supplementary Information

Supplementary Information.

## Data Availability

The datasets generated during and/or analyzed during the current study are available from the corresponding author on reasonable request.

## Code availability

The R scripts generated during the analysis of microarray datasets were submitted as a PDF file.
