# Supplementary material for: ITGA2, LAMB3, and LAMC2 may be the potential therapeutic targets in pancreatic ductal adenocarcinoma: an integrated bioinformatics analysis
Source: Sci Rep. 2021 May 18;11:10563. doi: 10.1038/s41598-021-90077-x (PMC8131351; doi:10.1038/s41598-021-90077-x)
Supplement: Supplementary file 1 — Supplementary Information. [file 41598_2021_90077_MOESM1_ESM.pdf]

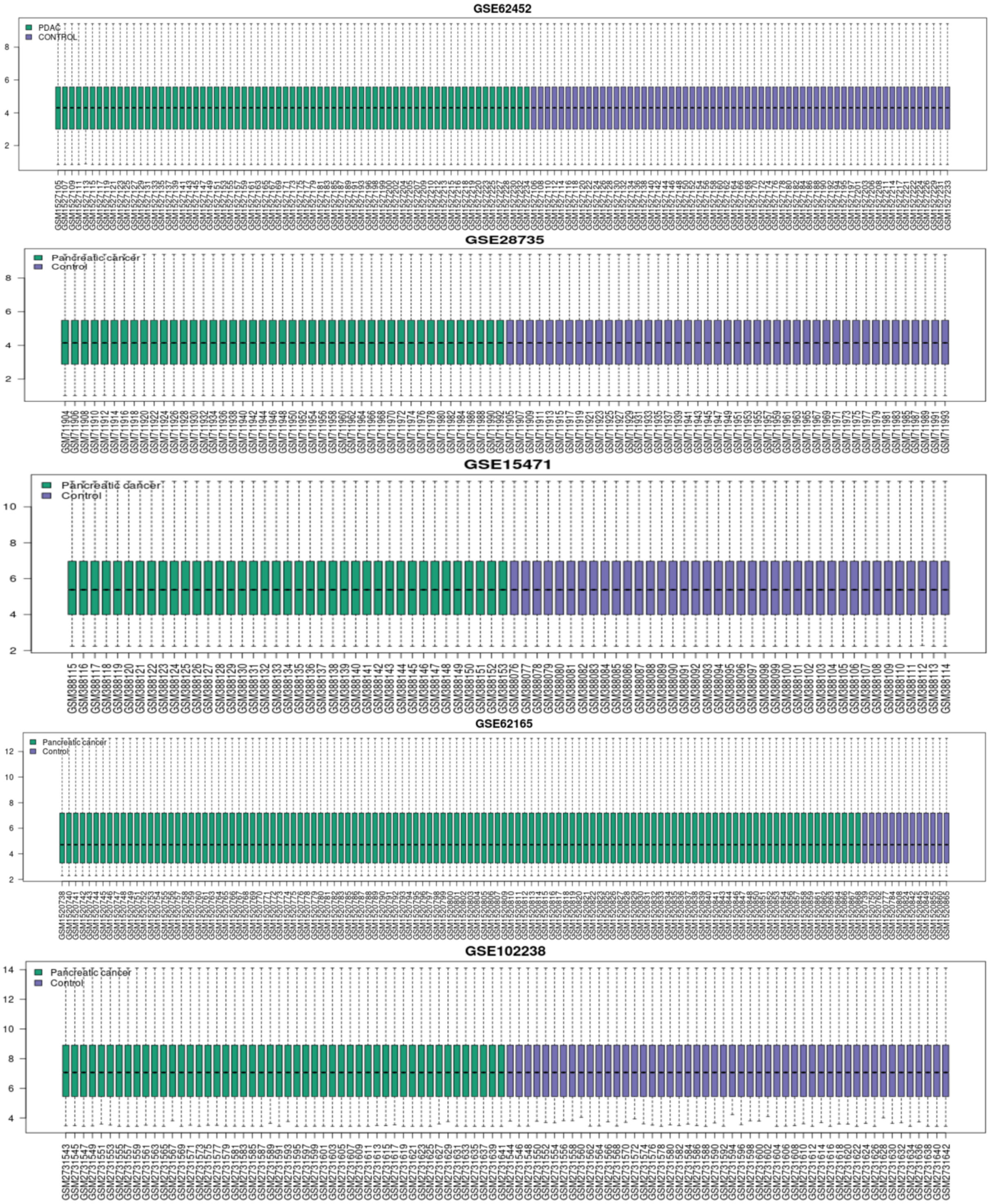

Supplementary Fig 1. Normalization of gene expression microarray datasets. The normalization of microarray datasets was performed by using the normalization module in the GEO2R package. The green and purple colors represent PDAC tissue and adjacent-non-cancerous tissue samples, respectively.

Table S1. Clinicopathological characteristics of the PDAC tissue samples in different microarray datasets

A, GEO accession: GSE62452, PDAC samples 69		
TNM stage, no. (%)	Grading, no. (%)	Survival status, no. (%)
Stage I, 4 (7.3%)	G1, 2 (2.9%)	succumbed, 49 (71.0%)
Stage IIA, 10 (12.2%)	G2, 35 (51.5%)	alive, 16 (23.2%)
Stage IIB, 35 (70.7%)	G3, 30 (44.1%)	N.A, (5.8%)
Stage III, 13 (9.8%)	G4, 1 (1.5%)	
Stage IV, 6 (8.8%)		
**B, GEO accession: GSE28735, PDAC samples 45**		
TNM stage, no. (%)	Grading, no. (%)	Survival status, no. (%)
Stage I, 1 (2%)	G1 and G2, 22 (49%)	succumbed, 29 (64.4%)
Stage II, 6 (14%)	G3 and G4, 23 (51%)	alive, 13 (28.9%)
Stage IIB, 21 (50%)		N.A, (6.7%)
Stage III, 9 (21%)		
Stage IV, 5 (12%)		
**C, GEO accession: GSE62165, PDAC samples 118**		
AJCC 7th edition, no. (%)	Grading, no. (%)	Survival status, no. (%)
≤ 2a, 38 (32.2%)	N.A	N.A
≥ 2b, 80 (67.8%)		
Grouped stage		
Early, 38 (32.2%)		
LNM, 62 (52.5%)		
Advanced, 18 (15.3%)		
**D, GEO accession: GSE102238, PDAC samples 50**		
TNM stage, no. (%)	Grading, no (%)	Survival status, no. (%)
T, 22 (44%)	G1 and G2, 30 (60%)	succumbed, 30 (60%)
N, 19 (38%)	G3, 20 (40%)	alive, 20 (40%)
M, 3 (6%)		

N.A, not available; AJCC, American Joint Committee on Cancer; LNM, lymph node metastasis

NOTE: clinicopathological details of the GSE15471 dataset was not available

R scripts

```
# Version info: R 3.2.3, Biobase 2.30.0, GEOquery 2.40.0, limma 3.26.8
################################################################
#   Differential expression analysis with limma
library(GEOquery)
library(limma)
library(umap)

# load series and platform data from GEO

gset <- getGEO("GSE62452", GSEMatrix =TRUE, AnnotGPL=TRUE)
if (length(gset) > 1) idx <- grep("GPL6244", attr(gset, "names")) else idx <- 1
```

```
gset <- gset[[idx]]

# make proper column names to match toptable
fvarLabels(gset) <- make.names(fvarLabels(gset))

# group membership for all samples
gsms <- paste0("01010101010101010101010101010101010101010101010101",
        "01010101010101010101010101010101010101011010001010",
        "010100100100100011010100101010")
sml <- strsplit(gsms, split="")[[1]]

# log2 transformation
ex <- exprs(gset)
qx <- as.numeric(quantile(ex, c(0., 0.25, 0.5, 0.75, 0.99, 1.0), na.rm=T))
LogC <- (qx[5] > 100) ||
          (qx[6]-qx[1] > 50 && qx[2] > 0)
if (LogC) { ex[which(ex <= 0)] <- NaN
  exprs(gset) <- log2(ex) }

exprs(gset) <- normalizeBetweenArrays(exprs(gset)) # normalize data

# assign samples to groups and set up design matrix
gs <- factor(sml)
groups <- make.names(c("PDAC","CONTROL"))
levels(gs) <- groups
gset$group <- gs
design <- model.matrix(~group + 0, gset)
colnames(design) <- levels(gs)

nall <- nrow(gset)
gset <- gset[complete.cases(exprs(gset)), ]

# calculate precision weights and show plot of mean-variance trend
v <- vooma(gset, design, plot=T)
# OR weights by group
# v <- voomaByGroup(gset, group=groups, design, plot=T, cex=0.1, pch=".", col=1:nlevels(gs))
v$genes <- fData(gset) # attach gene annotations

# fit linear model
fit  <- lmFit(v)

# set up contrasts of interest and recalculate model coefficients
cts <- c(paste(groups[1],"-",groups[2],sep=""))
cont.matrix <- makeContrasts(contrasts=cts, levels=design)
fit2 <- contrasts.fit(fit, cont.matrix)

# compute statistics and table of top significant genes
fit2 <- eBayes(fit2, 0.01)
tT <- topTable(fit2, adjust="fdr", sort.by="B", number=250)

tT <- subset(tT, select=c("ID","adj.P.Val","P.Value","t","B","logFC","Gene.symbol","Gene.title"))
write.table(tT, file=stdout(), row.names=F, sep="\t")

# Visualize and quality control test results.
# Build histogram of P-values for all genes. Normal test
# assumption is that most genes are not differentially expressed.
tT2 <- topTable(fit2, adjust="fdr", sort.by="B", number=Inf)
hist(tT2$adj.P.Val, col = "grey", border = "white", xlab = "P-adj",
  ylab = "Number of genes", main = "P-adj value distribution")
```

```
# summarize test results as "up", "down" or "not expressed"
dT <- decideTests(fit2, adjust.method="fdr", p.value=0.01)

# Venn diagram of results
vennDiagram(dT, circle.col=palette())

# create Q-Q plot for t-statistic
t.good <- which(!is.na(fit2$F)) # filter out bad probes
qqt(fit2$t[t.good], fit2$df.total[t.good], main="Moderated t statistic")

# volcano plot (log P-value vs log fold change)
colnames(fit2) # list contrast names
ct <- 1        # choose contrast of interest
volcanoplot(fit2, coef=ct, main=colnames(fit2)[ct], pch=20,
  highlight=length(which(dT[,ct]!=0)), names=rep('+', nrow(fit2)))

# MD plot (log fold change vs mean log expression)
# highlight statistically significant (p-adj < 0.01) probes
plotMD(fit2, column=ct, status=dT[,ct], legend=F, pch=20, cex=1)
abline(h=0)

################################################################
# General expression data analysis
ex <- exprs(gset)

# box-and-whisker plot
dev.new(width=3+ncol(gset)/6, height=5)
ord <- order(gs)  # order samples by group
palette(c("#1B9E77", "#7570B3", "#E7298A", "#E6AB02", "#D95F02",
         "#66A61E", "#A6761D", "#B32424", "#B324B3", "#666666"))
par(mar=c(7,4,2,1))
title <- paste ("GSE62452", "/", annotation(gset), sep ="")
boxplot(ex[,ord], boxwex=0.6, notch=T, main=title, outline=FALSE, las=2, col=gs[ord])
legend("topleft", groups, fill=palette(), bty="n")
dev.off()

# expression value distribution
par(mar=c(4,4,2,1))
title <- paste ("GSE62452", "/", annotation(gset), " value distribution", sep ="")
plotDensities(ex, group=gs, main=title, legend ="topright")

# UMAP plot (dimensionality reduction)
ex <- na.omit(ex) # eliminate rows with NAs
ex <- ex[!duplicated(ex), ]  # remove duplicates
ump <- umap(t(ex), n_neighbors = 15, random_state = 123)
par(mar=c(3,3,2,6), xpd=TRUE)
plot(ump$layout, main="UMAP plot, nbrs=15", xlab="", ylab="", col=gs, pch=20, cex=1.5)
legend("topright", inset=c(-0.15,0), legend=levels(gs), pch=20,
col=1:nlevels(gs), title="Group", pt.cex=1.5)
library("maptools")  # point labels without overlaps
pointLabel(ump$layout, labels = rownames(ump$layout), method="SANN", cex=0.6)



# Version info: R 3.2.3, Biobase 2.30.0, GEOquery 2.40.0, limma 3.26.8
################################################################
#   Differential expression analysis with limma
library(GEOquery)
library(limma)
library(umap)
```

```
# load series and platform data from GEO

gset <- getGEO("GSE28735", GSEMatrix =TRUE, AnnotGPL=TRUE)
if (length(gset) > 1) idx <- grep("GPL6244", attr(gset, "names")) else idx <- 1
gset <- gset[[idx]]

# make proper column names to match toptable
fvarLabels(gset) <- make.names(fvarLabels(gset))

# group membership for all samples
gsms <- paste0("01010101010101010101010101010101010101010101010101",
       "0101010101010101010101010101010101010101")
sml <- strsplit(gsms, split="")[[1]]

# log2 transformation
ex <- exprs(gset)
qx <- as.numeric(quantile(ex, c(0., 0.25, 0.5, 0.75, 0.99, 1.0), na.rm=T))
LogC <- (qx[5] > 100) ||
        (qx[6]-qx[1] > 50 && qx[2] > 0)
if (LogC) { ex[which(ex <= 0)] <- NaN
  exprs(gset) <- log2(ex) }

exprs(gset) <- normalizeBetweenArrays(exprs(gset)) # normalize data

# assign samples to groups and set up design matrix
gs <- factor(sml)
groups <- make.names(c("Pancreatic cancer","Control"))
levels(gs) <- groups
gset$group <- gs
design <- model.matrix(~group + 0, gset)
colnames(design) <- levels(gs)

nall <- nrow(gset)
gset <- gset[complete.cases(exprs(gset)), ]

# calculate precision weights and show plot of mean-variance trend
v <- vooma(gset, design, plot=T)
# OR weights by group
# v <- voomaByGroup(gset, group=groups, design, plot=T, cex=0.1, pch=".", col=1:nlevels(gs))
v$genes <- fData(gset) # attach gene annotations

# fit linear model
fit  <- lmFit(v)

# set up contrasts of interest and recalculate model coefficients
cts <- c(paste(groups[1],"-",groups[2],sep=""))
cont.matrix <- makeContrasts(contrasts=cts, levels=design)
fit2 <- contrasts.fit(fit, cont.matrix)

# compute statistics and table of top significant genes
fit2 <- eBayes(fit2, 0.01)
tT <- topTable(fit2, adjust="fdr", sort.by="B", number=250)

tT <- subset(tT, select=c("ID","adj.P.Val","P.Value","t","B","logFC","Gene.symbol","Gene.title"))
write.table(tT, file=stdout(), row.names=F, sep="\t")

# Visualize and quality control test results.
# Build histogram of P-values for all genes. Normal test
# assumption is that most genes are not differentially expressed.
```

```
tT2 <- topTable(fit2, adjust="fdr", sort.by="B", number=Inf)
hist(tT2$adj.P.Val, col = "grey", border = "white", xlab = "P-adj",
  ylab = "Number of genes", main = "P-adj value distribution")

# summarize test results as "up", "down" or "not expressed"
dT <- decideTests(fit2, adjust.method="fdr", p.value=0.01)

# Venn diagram of results
vennDiagram(dT, circle.col=palette())

# create Q-Q plot for t-statistic
t.good <- which(!is.na(fit2$F)) # filter out bad probes
qqt(fit2$t[t.good], fit2$df.total[t.good], main="Moderated t statistic")

# volcano plot (log P-value vs log fold change)
colnames(fit2) # list contrast names
ct <- 1        # choose contrast of interest
volcanoplot(fit2, coef=ct, main=colnames(fit2)[ct], pch=20,
  highlight=length(which(dT[,ct]!=0)), names=rep('+', nrow(fit2)))

# MD plot (log fold change vs mean log expression)
# highlight statistically significant (p-adj < 0.01) probes
plotMD(fit2, column=ct, status=dT[,ct], legend=F, pch=20, cex=1)
abline(h=0)

################################################################
# General expression data analysis
ex <- exprs(gset)

# box-and-whisker plot
dev.new(width=3+ncol(gset)/6, height=5)
ord <- order(gs)  # order samples by group
palette(c("#1B9E77", "#7570B3", "#E7298A", "#E6AB02", "#D95F02",
          "#66A61E", "#A6761D", "#B32424", "#B324B3", "#666666"))
par(mar=c(7,4,2,1))
title <- paste ("GSE28735", "/", annotation(gset), sep ="")
boxplot(ex[,ord], boxwex=0.6, notch=T, main=title, outline=FALSE, las=2, col=gs[ord])
legend("topleft", groups, fill=palette(), bty="n")
dev.off()

# expression value distribution
par(mar=c(4,4,2,1))
title <- paste ("GSE28735", "/", annotation(gset), " value distribution", sep ="")
plotDensities(ex, group=gs, main=title, legend ="topright")

# UMAP plot (dimensionality reduction)
ex <- na.omit(ex) # eliminate rows with NAs
ex <- ex[!duplicated(ex), ]  # remove duplicates
ump <- umap(t(ex), n_neighbors = 15, random_state = 123)
par(mar=c(3,3,2,6), xpd=TRUE)
plot(ump$layout, main="UMAP plot, nbrs=15", xlab="", ylab="", col=gs, pch=20, cex=1.5)
legend("topright", inset=c(-0.15,0), legend=levels(gs), pch=20,
col=1:nlevels(gs), title="Group", pt.cex=1.5)
library("maptools")  # point labels without overlaps
pointLabel(ump$layout, labels = rownames(ump$layout), method="SANN", cex=0.6)



# Version info: R 3.2.3, Biobase 2.30.0, GEOquery 2.40.0, limma 3.26.8
################################################################
```

```
#   Differential expression analysis with limma
library(GEOquery)
library(limma)
library(umap)

# load series and platform data from GEO

gset <- getGEO("GSE15471", GSEMatrix =TRUE, AnnotGPL=TRUE)
if (length(gset) > 1) idx <- grep("GPL570", attr(gset, "names")) else idx <- 1
gset <- gset[[idx]]

# make proper column names to match toptable
fvarLabels(gset) <- make.names(fvarLabels(gset))

# group membership for all samples
gsms <- paste0("11111111111111111111111111111111111111100000000000",
        "0000000000000000000000000000")
sml <- strsplit(gsms, split="")[[1]]

# log2 transformation
ex <- exprs(gset)
qx <- as.numeric(quantile(ex, c(0., 0.25, 0.5, 0.75, 0.99, 1.0), na.rm=T))
LogC <- (qx[5] > 100) ||
          (qx[6]-qx[1] > 50 && qx[2] > 0)
if (LogC) { ex[which(ex <= 0)] <- NaN
  exprs(gset) <- log2(ex) }

exprs(gset) <- normalizeBetweenArrays(exprs(gset)) # normalize data

# assign samples to groups and set up design matrix
gs <- factor(sml)
groups <- make.names(c("Pancreatic cancer","Control"))
levels(gs) <- groups
gset$group <- gs
design <- model.matrix(~group + 0, gset)
colnames(design) <- levels(gs)

nall <- nrow(gset)
gset <- gset[complete.cases(exprs(gset)), ]

# calculate precision weights and show plot of mean-variance trend
v <- vooma(gset, design, plot=T)
# OR weights by group
# v <- voomaByGroup(gset, group=groups, design, plot=T, cex=0.1, pch=".", col=1:nlevels(gs))
v$genes <- fData(gset) # attach gene annotations

# fit linear model
fit  <- lmFit(v)

# set up contrasts of interest and recalculate model coefficients
cts <- c(paste(groups[1],"-",groups[2],sep=""))
cont.matrix <- makeContrasts(contrasts=cts, levels=design)
fit2 <- contrasts.fit(fit, cont.matrix)

# compute statistics and table of top significant genes
fit2 <- eBayes(fit2, 0.01)
tT <- topTable(fit2, adjust="fdr", sort.by="B", number=250)

tT <- subset(tT, select=c("ID","adj.P.Val","P.Value","t","B","logFC","Gene.symbol","Gene.title"))
write.table(tT, file=stdout(), row.names=F, sep="\t")
```

```
# Visualize and quality control test results.
# Build histogram of P-values for all genes. Normal test
# assumption is that most genes are not differentially expressed.
tT2 <- topTable(fit2, adjust="fdr", sort.by="B", number=Inf)
hist(tT2$adj.P.Val, col = "grey", border = "white", xlab = "P-adj",
  ylab = "Number of genes", main = "P-adj value distribution")

# summarize test results as "up", "down" or "not expressed"
dT <- decideTests(fit2, adjust.method="fdr", p.value=0.01)

# Venn diagram of results
vennDiagram(dT, circle.col=palette())

# create Q-Q plot for t-statistic
t.good <- which(!is.na(fit2$F)) # filter out bad probes
qqt(fit2$t[t.good], fit2$df.total[t.good], main="Moderated t statistic")

# volcano plot (log P-value vs log fold change)
colnames(fit2) # list contrast names
ct <- 1        # choose contrast of interest
volcanoplot(fit2, coef=ct, main=colnames(fit2)[ct], pch=20,
  highlight=length(which(dT[,ct]!=0)), names=rep('+', nrow(fit2)))

# MD plot (log fold change vs mean log expression)
# highlight statistically significant (p-adj < 0.01) probes
plotMD(fit2, column=ct, status=dT[,ct], legend=F, pch=20, cex=1)
abline(h=0)

################################################################
# General expression data analysis
ex <- exprs(gset)

# box-and-whisker plot
dev.new(width=3+ncol(gset)/6, height=5)
ord <- order(gs)  # order samples by group
palette(c("#1B9E77", "#7570B3", "#E7298A", "#E6AB02", "#D95F02",
        "#66A61E", "#A6761D", "#B32424", "#B324B3", "#666666"))
par(mar=c(7,4,2,1))
title <- paste ("GSE15471", "/", annotation(gset), sep ="")
boxplot(ex[,ord], boxwex=0.6, notch=T, main=title, outline=FALSE, las=2, col=gs[ord])
legend("topleft", groups, fill=palette(), bty="n")
dev.off()

# expression value distribution
par(mar=c(4,4,2,1))
title <- paste ("GSE15471", "/", annotation(gset), " value distribution", sep ="")
plotDensities(ex, group=gs, main=title, legend ="topright")

# UMAP plot (dimensionality reduction)
ex <- na.omit(ex) # eliminate rows with NAs
ex <- ex[!duplicated(ex), ]  # remove duplicates
ump <- umap(t(ex), n_neighbors = 15, random_state = 123)
par(mar=c(3,3,2,6), xpd=TRUE)
plot(ump$layout, main="UMAP plot, nbrs=15", xlab="", ylab="", col=gs, pch=20, cex=1.5)
legend("topright", inset=c(-0.15,0), legend=levels(gs), pch=20,
col=1:nlevels(gs), title="Group", pt.cex=1.5)
library("maptools")  # point labels without overlaps
pointLabel(ump$layout, labels = rownames(ump$layout), method="SANN", cex=0.6)
```

```r
# Version info: R 3.2.3, Biobase 2.30.0, GEOquery 2.40.0, limma 3.26.8
################################################################
#   Differential expression analysis with limma
library(GEOquery)
library(limma)
library(umap)

# load series and platform data from GEO

gset <- getGEO("GSE62165", GSEMatrix =TRUE, AnnotGPL=FALSE)
if (length(gset) > 1) idx <- grep("GPL13667", attr(gset, "names")) else idx <- 1
gset <- gset[[idx]]

# make proper column names to match toptable
fvarLabels(gset) <- make.names(fvarLabels(gset))

# group membership for all samples
gsms <- paste0("010000000000010000000000010000000000000001000000100 0",
        "00000000000000000000100000000000000010000000000000",
        "0000100100010000010000100001000")
sml <- strsplit(gsms, split="")[[1]]

# log2 transformation
ex <- exprs(gset)
qx <- as.numeric(quantile(ex, c(0., 0.25, 0.5, 0.75, 0.99, 1.0), na.rm=T))
LogC <- (qx[5] > 100) ||
          (qx[6]-qx[1] > 50 && qx[2] > 0)
if (LogC) { ex[which(ex <= 0)] <- NaN
  exprs(gset) <- log2(ex) }

exprs(gset) <- normalizeBetweenArrays(exprs(gset)) # normalize data

# assign samples to groups and set up design matrix
gs <- factor(sml)
groups <- make.names(c("Pancreatic cancer","Control"))
levels(gs) <- groups
gset$group <- gs
design <- model.matrix(~group + 0, gset)
colnames(design) <- levels(gs)

nall <- nrow(gset)
gset <- gset[complete.cases(exprs(gset)), ]

# calculate precision weights and show plot of mean-variance trend
v <- vooma(gset, design, plot=T)
# OR weights by group
# v <- voomaByGroup(gset, group=groups, design, plot=T, cex=0.1, pch=".", col=1:nlevels(gs))
v$genes <- fData(gset) # attach gene annotations

# fit linear model
fit  <- lmFit(v)

# set up contrasts of interest and recalculate model coefficients
cts <- c(paste(groups[1],"-",groups[2],sep=""))
cont.matrix <- makeContrasts(contrasts=cts, levels=design)
fit2 <- contrasts.fit(fit, cont.matrix)

# compute statistics and table of top significant genes
```

```
fit2 <- eBayes(fit2, 0.01)
tT <- topTable(fit2, adjust="fdr", sort.by="B", number=250)

tT <- subset(tT,
select=c("ID","adj.P.Val","P.Value","t","B","logFC","Gene.Title","Gene.Symbol","GB_LIST","Ensembl"))
write.table(tT, file=stdout(), row.names=F, sep="\t")

# Visualize and quality control test results.
# Build histogram of P-values for all genes. Normal test
# assumption is that most genes are not differentially expressed.
tT2 <- topTable(fit2, adjust="fdr", sort.by="B", number=Inf)
hist(tT2$adj.P.Val, col = "grey", border = "white", xlab = "P-adj",
  ylab = "Number of genes", main = "P-adj value distribution")

# summarize test results as "up", "down" or "not expressed"
dT <- decideTests(fit2, adjust.method="fdr", p.value=0.01)

# Venn diagram of results
vennDiagram(dT, circle.col=palette())

# create Q-Q plot for t-statistic
t.good <- which(!is.na(fit2$F)) # filter out bad probes
qqt(fit2$t[t.good], fit2$df.total[t.good], main="Moderated t statistic")

# volcano plot (log P-value vs log fold change)
colnames(fit2) # list contrast names
ct <- 1        # choose contrast of interest
volcanoplot(fit2, coef=ct, main=colnames(fit2)[ct], pch=20,
  highlight=length(which(dT[,ct]!=0)), names=rep('+', nrow(fit2)))

# MD plot (log fold change vs mean log expression)
# highlight statistically significant (p-adj < 0.01) probes
plotMD(fit2, column=ct, status=dT[,ct], legend=F, pch=20, cex=1)
abline(h=0)

################################################################
# General expression data analysis
ex <- exprs(gset)

# box-and-whisker plot
dev.new(width=3+ncol(gset)/6, height=5)
ord <- order(gs)  # order samples by group
palette(c("#1B9E77", "#7570B3", "#E7298A", "#E6AB02", "#D95F02",
         "#66A61E", "#A6761D", "#B32424", "#B324B3", "#666666"))
par(mar=c(7,4,2,1))
title <- paste ("GSE62165", "/", annotation(gset), sep ="")
boxplot(ex[,ord], boxwex=0.6, notch=T, main=title, outline=FALSE, las=2, col=gs[ord])
legend("topleft", groups, fill=palette(), bty="n")
dev.off()

# expression value distribution
par(mar=c(4,4,2,1))
title <- paste ("GSE62165", "/", annotation(gset), " value distribution", sep ="")
plotDensities(ex, group=gs, main=title, legend ="topright")

# UMAP plot (dimensionality reduction)
ex <- na.omit(ex) # eliminate rows with NAs
ex <- ex[!duplicated(ex), ]  # remove duplicates
ump <- umap(t(ex), n_neighbors = 15, random_state = 123)
par(mar=c(3,3,2,6), xpd=TRUE)
```

```
plot(ump$layout, main="UMAP plot, nbrs=15", xlab="", ylab="", col=gs, pch=20, cex=1.5)
legend("topright", inset=c(-0.15,0), legend=levels(gs), pch=20,
col=1:nlevels(gs), title="Group", pt.cex=1.5)
library("maptools")  # point labels without overlaps
pointLabel(ump$layout, labels = rownames(ump$layout), method="SANN", cex=0.6)



# Version info: R 3.2.3, Biobase 2.30.0, GEOquery 2.40.0, limma 3.26.8
################################################################
#   Differential expression analysis with limma
library(GEOquery)
library(limma)
library(umap)

# load series and platform data from GEO

gset <- getGEO("GSE102238", GSEMatrix =TRUE, AnnotGPL=FALSE)
if (length(gset) > 1) idx <- grep("GPL19072", attr(gset, "names")) else idx <- 1
gset <- gset[[idx]]

# make proper column names to match toptable
fvarLabels(gset) <- make.names(fvarLabels(gset))

# group membership for all samples
gsms <- paste0("01010101010101010101010101010101010101010101010101010101",
        "01010101010101010101010101010101010101010101010101010101")
sml <- strsplit(gsms, split="")[[1]]

# log2 transformation
ex <- exprs(gset)
qx <- as.numeric(quantile(ex, c(0., 0.25, 0.5, 0.75, 0.99, 1.0), na.rm=T))
LogC <- (qx[5] > 100) ||
          (qx[6]-qx[1] > 50 && qx[2] > 0)
if (LogC) { ex[which(ex <= 0)] <- NaN
  exprs(gset) <- log2(ex) }

exprs(gset) <- normalizeBetweenArrays(exprs(gset)) # normalize data

# assign samples to groups and set up design matrix
gs <- factor(sml)
groups <- make.names(c("Pancreatic cancer","Control"))
levels(gs) <- groups
gset$group <- gs
design <- model.matrix(~group + 0, gset)
colnames(design) <- levels(gs)

nall <- nrow(gset)
gset <- gset[complete.cases(exprs(gset)), ]

# calculate precision weights and show plot of mean-variance trend
v <- vooma(gset, design, plot=T)
# OR weights by group
# v <- voomaByGroup(gset, group=groups, design, plot=T, cex=0.1, pch=".", col=1:nlevels(gs))
v$genes <- fData(gset) # attach gene annotations

# fit linear model
fit  <- lmFit(v)

# set up contrasts of interest and recalculate model coefficients
```

```
cts <- c(paste(groups[1],"-",groups[2],sep=""))
cont.matrix <- makeContrasts(contrasts=cts, levels=design)
fit2 <- contrasts.fit(fit, cont.matrix)

# compute statistics and table of top significant genes
fit2 <- eBayes(fit2, 0.01)
tT <- topTable(fit2, adjust="fdr", sort.by="B", number=250)

tT <- subset(tT,
select=c("ID","adj.P.Val","P.Value","t","B","logFC","SPOT_ID","CONTROL_TYPE","CHROMOSOMAL_L
OCATION","SEQUENCE"))
write.table(tT, file=stdout(), row.names=F, sep="\t")

# Visualize and quality control test results.
# Build histogram of P-values for all genes. Normal test
# assumption is that most genes are not differentially expressed.
tT2 <- topTable(fit2, adjust="fdr", sort.by="B", number=Inf)
hist(tT2$adj.P.Val, col = "grey", border = "white", xlab = "P-adj",
  ylab = "Number of genes", main = "P-adj value distribution")

# summarize test results as "up", "down" or "not expressed"
dT <- decideTests(fit2, adjust.method="fdr", p.value=0.01)

# Venn diagram of results
vennDiagram(dT, circle.col=palette())

# create Q-Q plot for t-statistic
t.good <- which(!is.na(fit2$F)) # filter out bad probes
qqt(fit2$t[t.good], fit2$df.total[t.good], main="Moderated t statistic")

# volcano plot (log P-value vs log fold change)
colnames(fit2) # list contrast names
ct <- 1        # choose contrast of interest
volcanoplot(fit2, coef=ct, main=colnames(fit2)[ct], pch=20,
  highlight=length(which(dT[,ct]!=0)), names=rep('+', nrow(fit2)))

# MD plot (log fold change vs mean log expression)
# highlight statistically significant (p-adj < 0.01) probes
plotMD(fit2, column=ct, status=dT[,ct], legend=F, pch=20, cex=1)
abline(h=0)

################################################################
# General expression data analysis
ex <- exprs(gset)

# box-and-whisker plot
dev.new(width=3+ncol(gset)/6, height=5)
ord <- order(gs)  # order samples by group
palette(c("#1B9E77", "#7570B3", "#E7298A", "#E6AB02", "#D95F02",
          "#66A61E", "#A6761D", "#B32424", "#B324B3", "#666666"))
par(mar=c(7,4,2,1))
title <- paste ("GSE102238", "/", annotation(gset), sep ="")
boxplot(ex[,ord], boxwex=0.6, notch=T, main=title, outline=FALSE, las=2, col=gs[ord])
legend("topleft", groups, fill=palette(), bty="n")
dev.off()

# expression value distribution
par(mar=c(4,4,2,1))
title <- paste ("GSE102238", "/", annotation(gset), " value distribution", sep ="")
plotDensities(ex, group=gs, main=title, legend ="topright")
```

```
# UMAP plot (dimensionality reduction)
ex <- na.omit(ex) # eliminate rows with NAs
ex <- ex[!duplicated(ex), ]  # remove duplicates
ump <- umap(t(ex), n_neighbors = 15, random_state = 123)
par(mar=c(3,3,2,6), xpd=TRUE)
plot(ump$layout, main="UMAP plot, nbrs=15", xlab="", ylab="", col=gs, pch=20, cex=1.5)
legend("topright", inset=c(-0.15,0), legend=levels(gs), pch=20,
col=1:nlevels(gs), title="Group", pt.cex=1.5)
library("maptools")  # point labels without overlaps
pointLabel(ump$layout, labels = rownames(ump$layout), method="SANN", cex=0.6)
```
